# Microalbuminuria: the earliest clinical evidence of diabetic nephropathy among individuals with type II diabetes mellitus

**DOI:** 10.11604/pamj.2025.52.49.47002

**Published:** 2025-09-30

**Authors:** Memory Ngosa, Bwalya Bupe Bwalya, Vaseemraja Shaikh, Bislom Chikwanka Mweene, Lukundo Siame, Tuku Mwakyoma, Joyce Kukeng´a Mwangu, Benson Malambo Hamooya, Sepiso Kenias Masenga

**Affiliations:** 1 HAND Research Group, School of Medicine and Health Sciences, Mulungushi University, Livingstone, Zambia,; 2 Department of Economics, School of Social Sciences, Mulungushi University, Kabwe, Zambia,; 3 Demography and Population Studies Program, School of Public Health and Social Sciences, University of Witwatersrand, Johannesburg, South Africa,; 4 Department of Cardiovascular Science and Metabolic Diseases, Livingstone Center for Prevention and Translational Science, Livingstone, Zambia

**Keywords:** Microalbuminuria, type 2 diabetes mellitus, end-stage kidney disease, diabetes kidney disease, hypertension

## To the editors of the Pan African Medical Journal

Diabetic nephropathy or diabetic kidney disease (DKD) is a serious complication of type 2 diabetes mellitus (T2DM), leading to conditions such as end-stage renal disease, heart failure, and early mortality, especially in African populations [1]. A key early marker of DKD is microalbuminuria, which indicates kidney damage but is undetectable by standard urine dipstick [2]. Early detection through regular screening is vital, as microalbuminuria can be reversed with timely treatment and lifestyle changes [3]. Despite recommendations for annual screening in T2DM patients, detection is often missed due to limited awareness and reliance on insensitive routine tests [4]. Diabetic Kidney disease may manifest as a sudden decline in kidney function, characterized by an increase in serum creatinine levels or a decrease in urine output [5]. Monitoring microalbumin levels helps identify individuals who may be at risk before severe kidney damage occurs. The primary objective of this study was to identify the factors associated with diabetic nephropathy among patients with type 2 diabetes mellitus (T2DM).

In this cross-sectional study, we included all patients aged 18 years or older, regardless of gender. The target population comprised persons with T2DM diagnosed as having a fasting blood glucose of ≥7.0 mmol/L and a history of using antidiabetic medication, and were being managed by physicians at Livingstone University Teaching Hospital (LUTH) in Livingstone Town, Zambia, from April to July 2023. For data collection, structured questionnaires were used to collect sociodemographic and clinical data. Urine samples were collected for laboratory tests to assess the presence of microalbuminuria. Microalbuminuria was the outcome variable defined according to the American diabetes association using a 24-hour collection technique as urine albumin concentration of 30 to 299 mg per 24 hours. We used SPSS version 22 software to analyze the data. Descriptive statistics, including medians, percentages, and frequency distributions, were used to describe the data and estimate the proportions of individuals with microalbuminuria. Ethical approval was sought from the Mulungushi University School of Medicine and Health Sciences Research Ethics Committee (MUSoMHSREC) (assurance no. FWA0002888 IRB00012281 of IORG0010344) on 15^th^ February 2023.

The median age of the participants was 55 years old (Interquartile range (IQR) (47.0 - 67.0), with the majority being female (n=56, 69.1%) and married (n=52, 64.2%). The majority of the participants attained a secondary level of education (n=29, 35.8%), were unemployed (n=65, 80.2%), and hypertensive (n=42, 51.9%). Participants with microalbuminuria were older (64, p<0.014), with longer diabetes duration (108 vs 60, p<0.035), and a history of being hospitalized at least once a year (1 vs 0, p<0.009). Concerning the clinical factors, participants with microalbuminuria had a slightly higher pulse pressure (57 vs 51, p<0.008), lower waist circumference (90 vs. 93.5, p<0.007), and a higher average vibration perception threshold (21 vs. 11.4, p<0.007). Participants with microalbuminuria had a slightly lower Urine specific gravity (1.02 vs. 1.03, p<0.025) (Table 1). At univariable analysis, a unit increase in the age of the patient had 6% odds of having microalbuminuria (odds ratio (OR); 0.06, 95% confidence interval (CI), (1.01 - 1.12, p<0.019) (Table 1). For every increase in the duration of T2DM (months), the odds of having microalbuminuria increased by 1% (AOR; 1.01(1.003 - 1.02, p<0.008). A unit increase in pulse pressure increased odds of having microalbuminuria (OR: 1.03 (1.01 - 1.06), p<0.020). An increase in Average vibration perception threshold had 1% odds of causing microalbuminuria (OR: 1.05 (1.01 - 1.09), p<0.026) (Table 1).

**Table 1:** sociodemographic, clinical characteristics, and factors associated with T2DM participants (N=81) screened for microalbuminuria at Livingstone University Teaching Hospital, Zambia (April-July 2023)

Variables	Frequency or median (Percentage or IQR)	Microalbuminuria		P-value
		Yes, (n= 19, 23.8%)	No, (n=61, 76.2%)	
**Age, years**	55 (12.31)	64 (54 - 69)	51 (46 - 63)	0.014
**Sex**				
Male	25 (30.9%)	4 (15.4%)	20 (76.9%)	0.069
Female	56 (69.1%)	15 (26.8%)	41 (73.2%)	
**Marital status**				
Married	52 (64.6%)	40 (75.5%)	11 (20.8%)	0.473
Unmarried	29 (35.4%)	21 (72.4%)	8 (27.6%)	
**Highest level of education**				
None	28 (34.6%)	7 (25%)	21 (75%)	0.791
Primary	3 (3.7%)	0 (0%)	3 (100%)	
Secondary	29 (35.8%)	6 (20.7%)	22 (75.9%)	
Tertiary	21 (25.9%)	6 (28.6%)	15 (71.4%)	
**Hypertension**				
Yes	42 (51.9%)	12 (29.3%)	29 (70.7%)	0.234
No	39 (48.1%)	7 (17.9%)	32 (82.1%)	
**Diabetes duration (months)**	60 (36 - 120)	108 (46 - 210)	60 (36 -108)	0.035
**No of hospital admissions**	1 (0-2)	1.0 (1.0 - 3.3)	0.0 (0.0 - 1.5)	0.009
**Systolic BP**	137 (126 -157)	142 (132 - 160)	134 (125 - 155.50)	0.107
**Diastolic BP**	85 (78.5 - 95.5)	85 (79 - 93)	85 (77 - 94)	0.713
**Pulse pressure**	53 (43.50 - 65)	57 (47 - 81)	51 (40 - 64)	0.008
**Ave vibration perception threshold**	14 (7.65 - 23.78)	21 (12.85 - 30.40)	11.40 (7.03 - 21.90)	0.007
**HbA1C**	9.53 (2.76)	7.74 (7.49 - 11.02)	9.56 (7.48 - 11.66)	0.345
**Hs-CRP (mg/L)**	2.54 (2.50 - 7.18)	2.50 (2.50 - 7.67)	2.68 (2.50 - 7.18)	0.365
**Specific gravity**	1.02 (1.02 - 1.03)	1.02 (1.02 - 1.03)	1.03 (1.02 - 1.03)	0.025
	OR (95%CI)	P-value	AOR (95%CI)	P-value
**Age, years**	0.06 (1.01 - 1.12)	0.019	1.04 (0.977 - 1.109)	0.214
**Sex**				
**Male**	0.547 (0.16 - 1.86)	0.334	0.26 (0.05 - 1.398)	0.117
**Female**	Ref			
**Diabetes duration (months)**	1.01 (1.003 - 1.02)	0.008	1.010 (1.002 - 1.019)	0.016
**No. of hospital admissions**	1.05 (1.91 - 1.21)	0.523		
**Pulse pressure**	1.03 (1.01 - 1.06)	0.020	1.01 (0.979 - 1.040)	0.564
**Ave. vibration perception threshold**	1.05 (1.01 - 1.09)	0.026	1.02 (0.968 - 1.066)	0.531
**Waist circumference (cm)**	1.00 (0.98 - 1.02)	0.883		
**Urine specific gravity**	5.23 (0.00 - 6111.26)	0.646		
**Plasma urea (mg/L)**	1.23 (0.98 - 1.53)	0.070		

BP: blood pressure, BMI: body mass index, HbA1C: glycated hemoglobin, ABI: ankle-brachial index, Hs-CRP: high-sensitivity C-reactive protein, Ave: average, cm: centimeters OR: odds ratio, AOR: adjusted odds ratio, CI: confidence interval

In multivariable analysis, diabetes duration was the only factor associated with microalbuminuria (adjusted odds ratio (AOR): 1.010, (Cl), 1.00, 1.01, p<0.016). Age, sex, number of hospital admissions, pulse pressure, average vibration perception threshold, waist circumference, urine specific gravity, and plasma urea were not associated with microalbuminuria (Table 1). The prevalence of microalbuminuria among T2DM was 23.8% (n=19), with only the duration of diabetes as the only significant factor associated with it. The global prevalence of microalbuminuria ranges between 7.8% to 39% among T2DM patients, while in Africa, an overall prevalence of 37.1% was recorded [6]. The prevalence recorded in our findings is consistent with a study conducted by Sana *et al*. reporting a high prevalence of 25.6% [7]. Contrary to our findings, a study conducted in Cameroon reported a lower prevalence of 14.2% [8]. Similarly, a study conducted in Tanzania, a country in the same region as Zambia, reported a lower prevalence rate varying significantly, with reports indicating rates as low as 9.8% [9]. In our findings, the high prevalence could be due to a lack of routine check-ups for microalbuminuria among T2DM patients who come for diabetic clinics, despite microalbuminuria remaining the most reliable indicator of diabetic nephropathy among diabetic patients. The microalbuminuria test is rarely conducted at our hospital in patients living with T2DM. Comorbidities, including hypertension, which worsen systemic vasculopathy and other microvascular consequences such as retinopathy, nephropathy, and cardiovascular diseases, are some of the factors contributing to the high prevalence [6].

In our study, we found a correlation between microalbuminuria and the duration of T2DM. The duration of diabetes is a significant factor in the onset and advancement of diabetic nephropathy, and progression can be slowed or reversed by strict control of hyperglycemia. Gui *et al*. reported that diabetes duration and the pathophysiological alterations in diabetic kidney disease are tightly related [10]. Chronic hyperglycemia causes a number of metabolic problems, such as reactive oxygen species (ROS) generation and inflammatory pathway activation, both of which increase the risk of kidney damage [11].

In our study, the positive correlation highlights poor glycemic control, which is common in our setting where diabetic medication is expensive and diabetic routine tests (HbA1c, kidney function tests, vibration perception) are rarely done in diabetic clinics due to limited resources [12]. In addition, shortages of specialists in diabetes care have been highlighted as challenges in low-income countries. In our study, the duration of diabetes was a significant factor in the onset and advancement of diabetic kidney disease, although early detection of Microalbuminuria remains a challenge, necessitating efforts to address these challenges to prevent chronic kidney disease, which is believed to be prevalent in Zambia.

While the study offered insights into the prevalence and associated factors of microalbuminuria, it had limitations, including a small sample size and a cross-sectional design that limited causal inference. The absence of a control group, a defined timeframe, and a narrow geographic scope also restricted the ability to account for cultural and socioeconomic differences that may influence microalbuminuria management and outcomes.

## Conclusion

The study found a significant link between diabetes duration and microalbuminuria. Regular check-ups in diabetic clinics could help reduce the risk of acute kidney injury and progression to kidney disease among T2DM patients. Further research with larger sample sizes and longitudinal designs is needed to better understand the risk factors and improve prevention and treatment strategies in this setting.

## References

[1] Azagew AW, Beko ZW, Mekonnen CK (2024). Determinants of diabetic nephropathy among diabetic patients in Ethiopia: Systematic review and meta-analysis. PLoS One.

[2] Vaidya SR, Aeddula NR (2024). Chronic Kidney Disease. StatPearls.

[3] Kitada M, Kanasaki K, Koya D (2014). Clinical therapeutic strategies for early stage of diabetic kidney disease. World J Diabetes.

[4] Christofides EA, Desai N (2021). Optimal Early Diagnosis and Monitoring of Diabetic Kidney Disease in Type 2 Diabetes Mellitus: Addressing the Barriers to Albuminuria Testing. J Prim Care Community Health.

[5] Turgut F, Awad AS, Abdel-Rahman EM (2023). Acute Kidney Injury: Medical Causes and Pathogenesis. J Clin Med.

[6] Mohammed O, Alemayehu E, Bisetegn H, Debash H, Gedefie A, Ebrahim H (2023). Prevalence of Microalbuminuria Among Diabetes Patients in Africa: A Systematic Review and Meta-Analysis. Diabetes Metab Syndr Obes.

[7] Sana MA, Chaudhry M, Malik A, Iqbal N, Zakiuddin A, Abdullah M (2020). Prevalence of Microalbuminuria in Type 2 Diabetes Mellitus. Cureus.

[8] Efundem NT, Assob JCN, Feteh VF, Choukem SP (2017). Prevalence and associations of microalbuminuria in proteinuria-negative patients with type 2 diabetes in two regional hospitals in Cameroon: a cross-sectional study. BMC Res Notes.

[9] Al-Maskari F, El-Sadig M, Obineche E (2008). Prevalence and determinants of microalbuminuria among diabetic patients in the United Arab Emirates. BMC Nephrol.

[10] Gui Y, Palanza Z, Fu H, Zhou D (2023). Acute kidney injury in diabetes mellitus: epidemiology, diagnostic and therapeutic concepts. FASEB J.

[11] Amorim RG, Guedes GD, Vasconcelos SM, Santos JC (2019). Kidney Disease in Diabetes Mellitus: Cross-Linking between Hyperglycemia, Redox Imbalance and Inflammation. Arq Bras Cardiol.

[12] Abera RG, Demesse ES, Boko WD (2022). Evaluation of glycemic control and related factors among outpatients with type 2 diabetes at Tikur Anbessa Specialized Hospital, Addis Ababa, Ethiopia: a cross-sectional study. BMC Endocr Disord.

